# Negative Pressure Wound Therapy in an Immunocompromised Child: A Case Report From a Resource‐Limited Setting in Ghana

**DOI:** 10.1002/ccr3.71846

**Published:** 2026-01-14

**Authors:** Matilda Mawusi Kodjo, Kennedy Kofi Kru, Belinda Dziedzorm Korley, Cynthia Tetteh, Wise Awunyo, Agani Afaya

**Affiliations:** ^1^ Department of Nursing University of Health and Allied Sciences Ho Ghana; ^2^ Ho Polyclinic Volta Region Ghana; ^3^ Keta Municipal Hospital Volta Region Ghana; ^4^ Department of Public Health Nursing University of Health and Allied Sciences Ho Ghana

**Keywords:** immunocompromised child, improvised vacuum‐assisted closure device, negative pressure wound therapy, septic wound

## Abstract

An 8‐year‐old immunocompromised girl with a large septic wound on the left thigh was admitted to the facility after the failure of home treatment by the family. The child was generally stable aside from the difficulty in walking on the left leg. An improvised vacuum‐assisted closure device was used to promote wound healing, as the actual device was not available. The wound experienced massive and impressive improvement within the first week of admission. Healthy and beautiful granulation tissues filled up the depth of the wound, closing it up by the third day of wound dressing. This case illustrates the possibility of healing large wounds faster than expected using negative pressure wound therapy, where the patient's immune status does not affect the healing process. This can be employed to reduce patients' length of hospital stay as well as reduce the workload on staff.

## Key Clinical Message

Negative pressure wound therapy enhances rapid wound healing, regardless of wound size and immune status, in children. This method has proven to be effective even with improvised items.

## 1 Introduction

Negative pressure wound therapy (NPWT), also known as the vacuum‐assisted closure technique, is effective for infectious wounds at all sites of the body [1, 2, 3]. The NPWT device is uncommon in developing countries, including Ghana, because of its high cost. Evidence has shown that this method of wound management is very effective for all wound types, promotes early wound healing, and reduces hospital stays as compared to the traditional ways of wound dressing [4]. The device is hardly available in hospitals and clinics where wounds are managed daily. A study conducted outside Ghana demonstrated the use of readily available items that can be used in place of the standard machine [5].

This was a case report on a large septic wound that healed quickly using the improvised items for a vacuum‐assisted closure device (VAC) for NPWT. The wound healed in a few days, despite the child being immunocompromised, which was even faster than the extended time it would have taken with the traditional method. NPWT is not commonly used in Ghana, and only one study evaluated the effectiveness of NPWT in adult patients. A study conducted in Ghana by Akpaloo et al. [6] used the actual VAC machine, which was very effective in the healing of wounds. The study excluded children; however, De Jesus et al. [7] established that NPWT is safe for the pediatric population, including neonates.

The patient in this report was a child who was treated with an improvised VAC device, demonstrating that NPWT was also effective for use in children. There are limited studies in the Volta Region that have reported the use of NPWT generally, not to mention its use in children, as the hospitals in the region do not have a VAC device for wound dressing.

## 2 Case History/Examination

On 27th January 2021, Patient J.T., an 8‐year‐old girl, was brought to a health facility in the Volta Region of Ghana on account of a wound on the left thigh. She was brought in by her mother, who assisted her in walking. According to the child's mother, the child had a boil on her thigh for about 5 weeks. The mother applied a local topical cream to the boil, which subsequently burst into a large wound for about a week before seeking medical attention at the facility. The child lives with her parents and two younger siblings in a suburb of Ho in the Volta Region of Ghana.

On observation, she was fully conscious, not in any form of respiratory distress. She was afebrile and anicteric but very pale. The child was in pain with an arthralgic gait. Her vital signs (Temperature, Pulse, and Respiration) were within normal ranges, and she weighed 20 kg. The wound was situated on the left upper thigh, near the groin. It was extensively sloughy and greenish, measuring approximately 8 cm in width and 3 cm in depth. The child was generally stable, aside from the pain in her left leg. She had a few scars from healed rashes on her lower limbs, and all other systems were unremarkable.

## 3 Differential Diagnosis

The child was admitted to the children's ward, diagnosed with a septic wound due to the state of the wound, and suspected of retroviral infection due to some rashes on her scalp as well as old scars on her limbs.

## 4 Investigation

Blood samples were taken and sent to the laboratory for analysis: complete blood count and a retroviral test to rule out infections. The results of the blood work indicated a white blood cell count of 11 × 10^9^/L, hemoglobin level of 7.7 g/dL, sickling‐negative, and retrovirus‐positive.

## 5 Treatment

The patient was treated with intravenous (IV) clindamycin for 5 days, IV paracetamol, IV 4.3% dextrose in 0.18% normal saline, vitamin C, syrup zincovite, savlon, normal saline, and povidone solution were also prescribed for wound dressing. Nutritional counseling was done to ensure that the child's meals had rich amounts of protein, carbohydrates, and vitamins to facilitate the wound healing process. The antiretroviral therapy unit was also consulted to initiate treatment.

**FIGURE 1:**
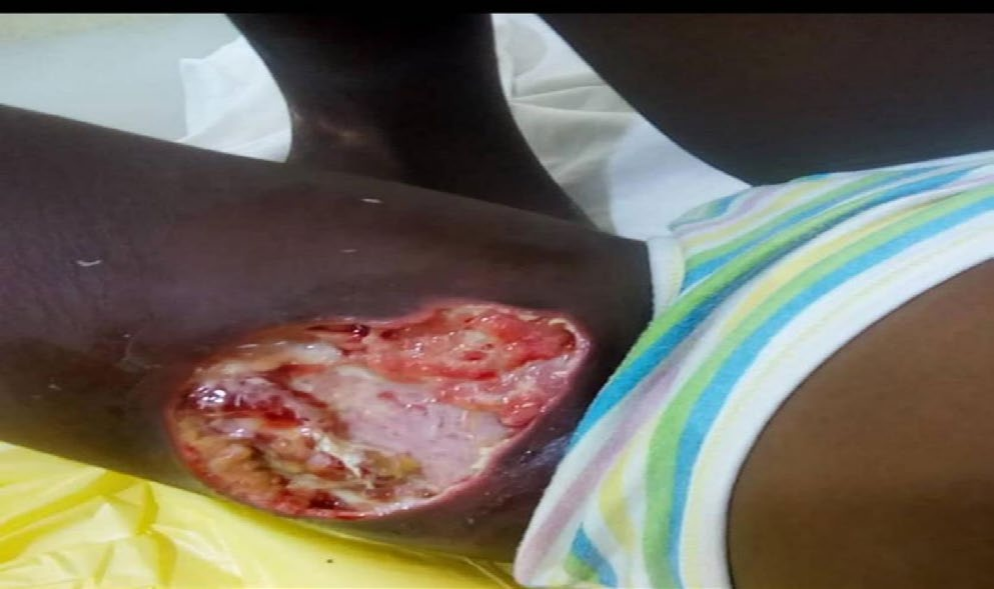
Wound after debridement 1st day of admission (28/1/21).

On January 28, 2021, Co‐trimoxazole (Septrin) was started for the child to manage the retroviral infection. Minor debridement was performed to clean the wound (Figure 1). The main antiretroviral medications were introduced later, after she recovered, to prevent immune reconstitution inflammatory syndrome (IRIS).

**FIGURE 2:**
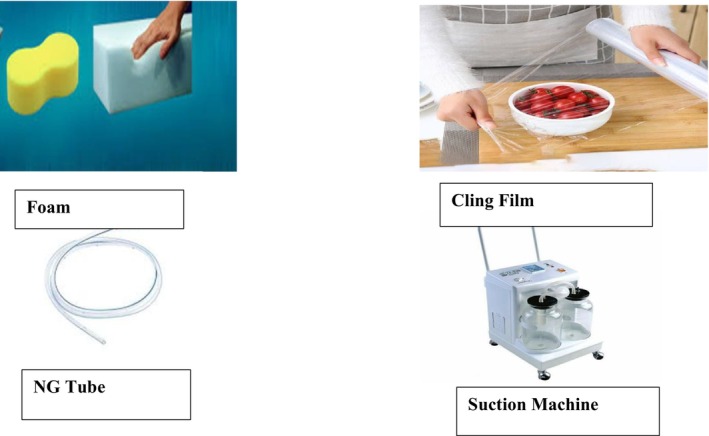
Items used for the improvised NPWT.

**FIGURE 3:**
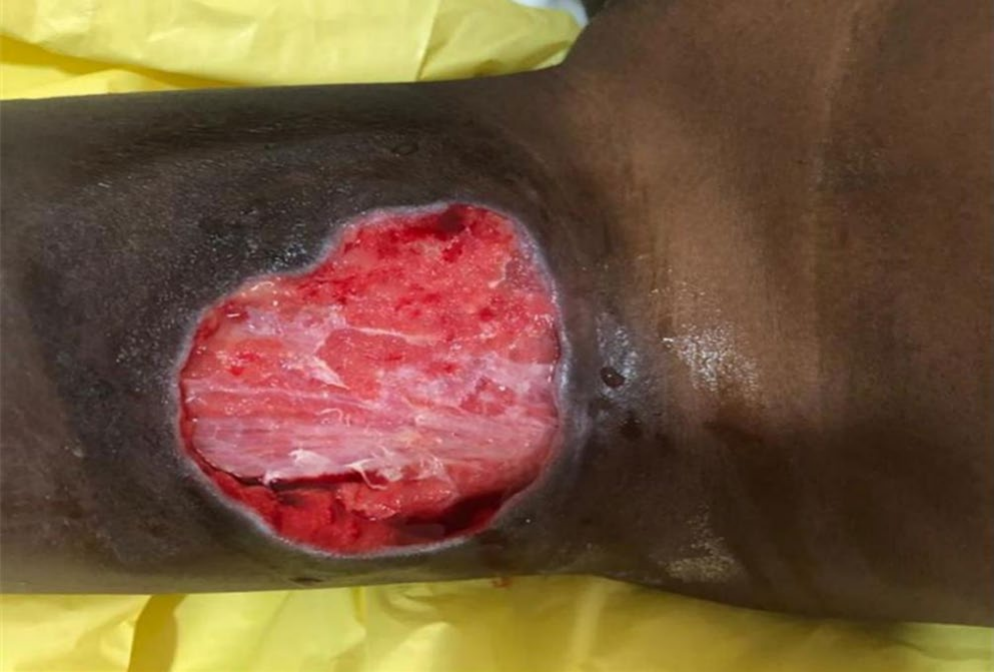
Beautiful granulation tissues on the 3rd day of admission (1/2/21).

The following day, January 29, an improvised NPWT was applied using the following items: a suction machine (set to a pressure of 120 mmHg), a nasogastric tube, domestic foam (cut to fit the wound size and sterilized), and cling film (Figure 2). During the wound dressing, the therapy was applied after the wound was cleaned with normal saline and covered with sterile gauze and povidone‐iodine solution. The NPWT system was assembled using readily available materials mentioned earlier in the absence of the original NPWT machine. The suction machine was set to deliver a negative pressure of 120 mmHg, and the nasogastric tube, which acted as a channel for wound exudate removal. One end of the nasogastric tube was connected to the suction machine, and the other end was inserted into the sterile domestic foam. The sterilized domestic foam, sized according to the patient's wound, was placed over the covered wound. An airtight seal was made with the cling film over the foam and tubing to maintain the negative‐pressure environment. The therapy was administered for 15 min every hour and documented accordingly for 3 days. The dressing was changed every other day. By the third day of treatment, granulation tissue had significantly closed the wound (Figure 3). The NPWT was discontinued, and the traditional dressing was resumed until discharge.

According to Normandin et al. [8], the NPWT works by removing excess fluid (which delays wound healing) in the wound through suctioning. It also controls swelling by reducing edema around the wound. The suctioning promotes blood flow to the wound site, and this increases oxygen and nutrients to repair worn‐out tissues. The wound edges are pulled together, which stimulates cell proliferation and granulation tissue formation, resulting in faster wound healing.

## 6 Outcome and Follow‐Up

**FIGURE 4:**
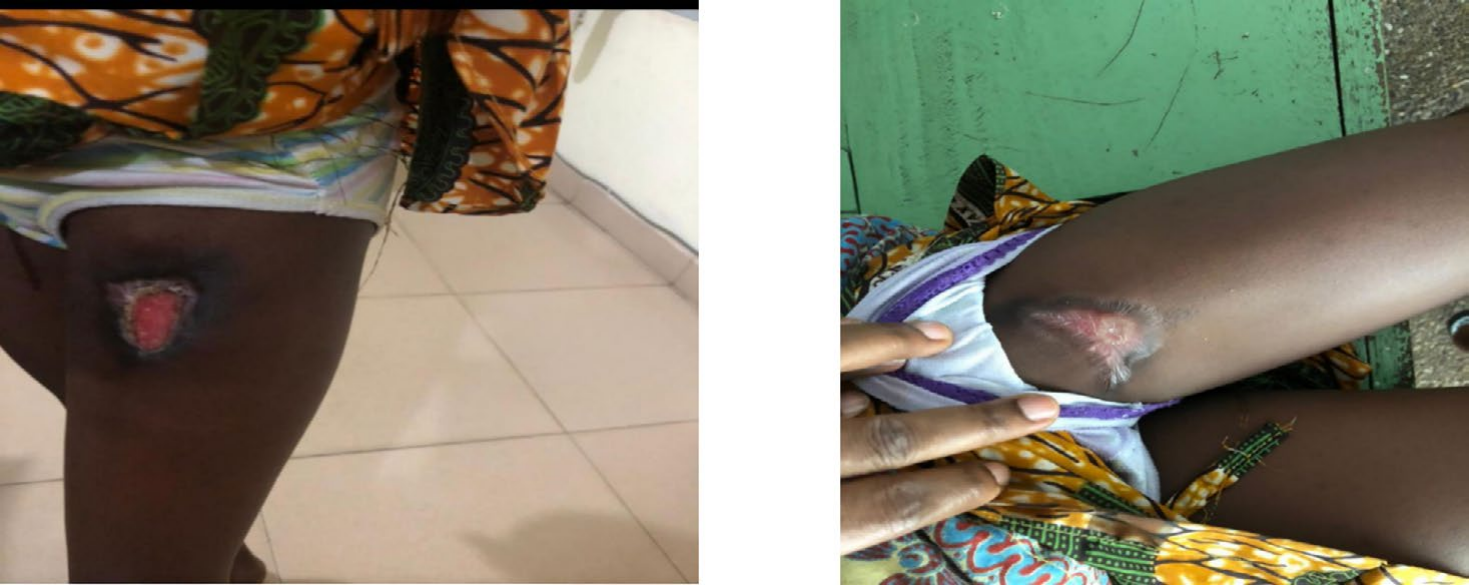
Wound completely healed 8th week 29/3/21.

The patient spent a total of 7 days in the hospital and was discharged on February 3, 2021, with instructions for wound dressing at a clinic near her home. At discharge, she regained normal gait, her hemoglobin level rose to 9.9 g/dL, and her white blood cell count was 6 × 10^9^/L. The family left feeling very happy and satisfied with the rapid healing process. They were also instructed to report to the facility for a review once a week for the first 2 weeks, and then once every fortnight until the wound was completely healed (Figure 4).

## 7 Discussion

NPWT is an essential treatment for complex wounds that are not healing effectively or are at high risk of nonhealing [9]. These complex wounds may be either acute or chronic. NPWT involves the application of sub‐atmospheric pressure to wound sites to reduce inflammatory exudate formation and actively promote the formation of granulation tissue [6]. Agarwal et al. [9] highlighted that NPWT decreases wound size and depth when applied, accelerates wound healing, and in turn lowers both costs and length of hospital stay.

NPWT is particularly recommended for acute wounds that cannot heal by primary intention, especially in cases involving risks such as potential or ongoing infection, skin tension, or swelling [4, 10]. According to Huang et al. [11] NPWT serves as an effective adjunctive treatment for managing complex wounds, promoting wound healing, and supporting skin integrity restoration. When utilized properly by trained healthcare professionals, NPWT contributes to improved clinical outcomes. Akpaloo et al. [6] further emphasized its effectiveness in their study conducted in Kumasi, Ghana.

Owing to the high cost of the VAC device, it is often unavailable in many healthcare facilities, particularly in low‐resource settings [5]. Kamamoto et al. [12] compared the effectiveness of a university‐made improvised VAC device developed by the University of São Paulo (USP) and found it to be as effective as the commercially available devices. It was also identified that the improvised device was more economical. The materials used in that study were similar to the ones used in this case report. Similarly, Gopal and Solomon [5] successfully used improvised items to administer NPWT for a gangrenous foot, substituting a Foley catheter and glove in place of the nasogastric tube and cling film used in this report. These improvised methods have all proven to be effective alternatives to the actual VAC devices.

Alhajj and Goyal [13] reported that traditional wound dressing with normal saline and povidone‐iodine typically takes several weeks for granulation tissue to fill a wound, depending on its size. Similarly, the Royal Children's Hospital [14] estimated granulation tissue formation to take approximately 3–4 weeks, varying with wound size. This process may be further prolonged in immunocompromised patients.

In this case, the patient's wound was fully covered with healthy granulation tissue by the third day of NPWT application, despite her immunocompromised status; a very impressive outcome. The patient's hospital stay was notably short (7 days) due to rapid wound improvement with NPWT, which aligns with Zens et al. [15], who found that NPWT reduces hospital stays and readmissions compared to traditional wound dressing methods. By the eighth week (29/03/2021), the patient's wound had completely healed, as confirmed during their follow‐up visit.

According to Huang et al. [11] and Zaver and Kankanalu [10], certain patient‐specific situations can make the application of NPWT unsafe. Cases involving exposed blood vessels or organs are contraindicated for NPWT to prevent the risk of serious bleeding. Additionally, NPWT is not recommended for non‐enteric or unexplored fistulas, as it may cause excessive fluid loss, leading to dehydration and electrolyte imbalances.

While some studies suggest that NPWT is generally unsuitable for cancer patients due to concerns about potential tumor growth and metastasis, Cai et al. [16] argued that it should not be universally contraindicated. Instead, each patient's condition should be individually assessed, with treatment decisions guided by available evidence.

NPWT, although highly effective in promoting wound healing, may present with some complications. Notably, among those complications are severe bleeding, severe pain, dehydration, skin irritation, and infection [6, 16].

Aside from moderate pain of 4/10 (numeric scale) during the therapy, the patient did not experience any other complications. The child's pain was managed at each session with non‐pharmacological efforts and analgesics.

## 8 Conclusion

NPWT has been effective in facilitating wound healing across various cases in both adults and children. Given the high cost of commercial NPWT devices, exploring alternative solutions is essential. Exploring its potential and enhancing existing improvised methods or developing cost‐effective versions, tailored to the Ghanaian context, could provide a more accessible and sustainable option for better wound management to improve patient outcomes.

## Author Contributions

**Matilda Mawusi Kodjo:** conceptualization, data curation, investigation, methodology, project administration, resources, writing – original draft. **Kennedy Kofi Kru:** investigation, writing – original draft. **Belinda Dziedzorm Korley:** investigation, writing – review and editing. **Cynthia Tetteh:** investigation, writing – review and editing. **Wise Awunyo:** investigation, writing – review and editing. **Agani Afaya:** investigation, supervision, writing – review and editing.

## Funding

The authors have nothing to report.

## Ethics Statement

The authors have nothing to report.

## Consent

Written informed consent has been obtained from the patient and the parent involved in this study.

## Conflicts of Interest

The authors declare no conflicts of interest.

## Data Availability

The data used in this article are available upon request from the authors.
